# Scene processing following damage to the ventromedial prefrontal cortex

**DOI:** 10.1097/WNR.0000000000001281

**Published:** 2019-07-04

**Authors:** Flavia De Luca, Cornelia McCormick, Elisa Ciaramelli, Eleanor A. Maguire

**Affiliations:** a Dipartimento di Psicologia, Centro studi e ricerche di Neuroscienze Cognitive, Università di Bologna, Bologna, Italy; b Wellcome Centre for Human Neuroimaging, UCL Queen Square Institute of Neurology, University College London, London, UK

**Keywords:** autobiographical memory, confabulation, episodic memory, future thinking, hippocampus, impossible scenes, scene construction, scene semantics, schema, ventromedial, prefrontal cortex

## Abstract

It has been suggested that the mental construction of scene imagery is a core process underpinning functions such as autobiographical memory, future thinking and spatial navigation. Damage to the ventromedial prefrontal cortex in humans can cause deficits in all of these cognitive domains. Moreover, it has also been reported that patients with ventromedial prefrontal cortex lesions are impaired at imagining fictitious scenes, although they seem able to describe specific scenes from autobiographical events. In general, not much is known about how ventromedial prefrontal cortex patients process scenes. Here, we deployed a recently-developed task to provide insights into this issue, which involved detecting either semantic (e.g. an elephant with butterflies for ears) or constructive (e.g. an endless staircase) violations in scene images. Identifying constructive violations typically provokes the formation of internal scene models in healthy control participants. We tested patients with bilateral ventromedial prefrontal cortex damage, brain-damaged control patients and healthy control participants. We found no evidence for statistically significant differences between the groups in detecting either type of violation. These results suggest that an intact ventromedial prefrontal cortex is not necessary for some aspects of scene processing, with implications for understanding its role in functions such as autobiographical memory and future thinking.

## Introduction

It has been suggested that the mental construction of scene imagery is a core process underpinning functions such as autobiographical memory, future thinking and spatial navigation [1,2]. A scene is defined as a naturalistic three-dimensional spatially coherent representation of the world typically populated by objects and viewed from an egocentric perspective [2,3]. The hippocampus in particular has been suggested to play a key role in supporting scene imagery. This is because patients with hippocampal damage struggle to imagine spatially coherent scenes, even when heavily cued [4], or when the construction of scenes is assessed implicitly [5]. McCormick *et al.* [6] recently confirmed that it is the constructive aspect of scene processing that seems to be particularly compromised by hippocampal lesions. They used a paradigm that involved detecting either semantic (e.g. an elephant with butterflies for ears) or spatial constructive (e.g. an endless staircase) violations in naturalistic scene images. Therefore, scenes could be semantically or constructively ‘possible’ or ‘impossible’. Healthy control participants indicated that they constructed flexible mental representations of the scenes in order to detect constructive, but not semantic, violations. Aligning with this finding, hippocampal-damaged patients were significantly impaired at deciding if scenes were constructively possible or impossible, but were unimpaired at making the semantic judgements.

Patients with bilateral ventromedial prefrontal cortex (vmPFC) damage also display a deficit in mentally constructing scene imagery when tested with relatively unconstrained cues, such as during the free recall of autobiographical memories [7], or the imagination of future and fictitious scenarios [7–9], and even when the construction of scenes is assessed implicitly [10]. However, vmPFC-lesioned patients are able to describe single scenes from autobiographical memories when heavily cued [11], suggesting their basic ability to generate scene imagery may be intact. vmPFC-damaged patients also have other cognitive deficits that seem to coalesce around a reduced ability to initiate endogenous processing. This led McCormick *et al*. [12] (see also Ref. 13) to propose that the vmPFC initiates the activation of schematic [14] and other knowledge in neocortex that is relevant for scene imagery while inhibiting elements that are irrelevant. This information is then conveyed to the hippocampus, which constructs the scene image.

Overall, however, little is known about the ability of patients with vmPFC lesions to process scenes. Therefore, in this study we examined how patients with bilateral vmPFC damage performed on McCormick *et al.’s* [6] possible/impossible scenes task. With the scene stimuli and the task demands acting as strong cues to guide endogenous (including constructive) processing, thus mitigating any initiation difficulties, and if their basic ability to construct scene imagery is preserved (given their intact hippocampi), then the vmPFC-damaged patients should be unimpaired. However, if they show deficits, this could suggest a more fundamental deficit in the generation of scene imagery, speaking against McCormick *et al.’s* [12] theory that the vmPFC is involved in the initiation of the scene construction process.

## Methods

### Participants

There were 34 participants. Eight were patients with bilateral vmPFC damage and 10 were ‘control patients’ with brain damage not involving vmPFC (or the medial temporal lobe). Brain damage in vmPFC patients was bilateral in all cases and resulted from the rupture of an anterior communicating artery aneurysm. In control patients, brain damage (left hemisphere: five cases; right hemisphere: five cases) was due to stroke (six cases), arteriovenous malformations (one case), intraparenchymal bleeding (one case), cerebral abscess (one case) or meningioma (one case). All patients were in a stable phase of health, and had no other diagnoses likely to affect cognition or interfere with participation in the study (e.g. significant psychiatric disease, alcohol abuse, history of cerebrovascular disease).

**Fig. 1:**
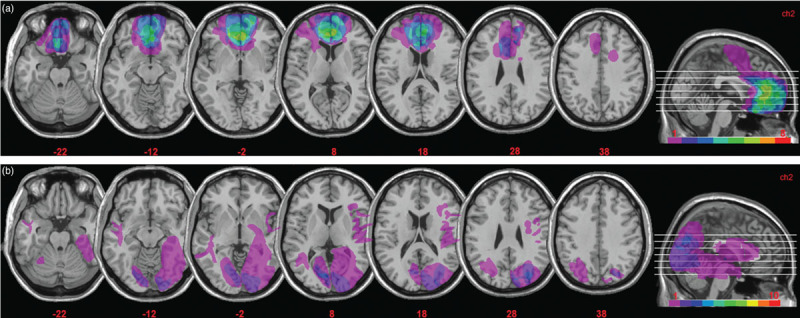
(a) Representative axial slices and cumulative midsagittal views of the standard Montreal Neurological Institute brain showing the extent of lesion overlap in the vmPFC patients. The white horizontal lines on the sagittal view are the positions of the axial slices, and the red numbers below the axial views are the x coordinates of each slice. The colour bar indicates the number of overlapping lesions. Maximal overlap occurred in BA 10, 11 and 32. The left hemisphere is on the left side. (b) Extent and overlap of brain lesions for the control patients. The figure represents the patients’ lesions projected on the same six axial slices of the standard Montreal Neurological Institute brain as shown in (a) above. Maximal overlap occurred in BA’s 17–19, 37. BA, Brodmann areas; vmPFC, ventromedial prefrontal cortex.

Lesions were derived from MRI or computerized tomography images, and were manually drawn by an expert neurologist (not involved in the study, and blind to task performance), or by one of the authors who was trained in manual segmentation. The same expert neurologist then verified all scans directly on each slice of the normalized T1-weighted template MRI scan from the Montreal Neurological Institute, approximately oriented to match Talairach space and distributed with MRIcro [15]. MRIcro software was used to estimate lesion volumes (in cm^3^) and generate lesion overlap images. Fig. 1a shows the extent and overlap of brain lesions in the vmPFC patients. The Brodmann areas (BAs) that were mainly affected were BA 10, BA 11, BA 24, BA 25, BA 32, with the region of maximal overlap occurring in BA 11 (M = 16.22 cm^3^, SD = 10.23), BA 10 (M = 9.42 cm^3^, SD = 7.80) and BA 32 (M = 6.71 cm^3^, SD = 5.22). One vmPFC patient had a very large lesion that extended to dorsal prefrontal cortex (BA 6 and BA 8). Excluding this patient from the analyses, however, did not alter the results. For the control patients (Fig. 1b), the areas mainly affected were BAs 17–19 (M = 13.77 cm^3^, SD = 19.42), BAs 20–22 and BA 37 (M = 4.49 cm^3^, SD = 10.21). There was no significant difference in lesion volume between vmPFC patients and control patients (46.27 vs. 24.22 cm^3^, *t* = 1.74, *P* = 0.10).

Table 1 summarizes the vmPFC and control patients’ neuropsychological profiles (and includes a brief description of the tests used). In general the vmPFC and control patients’ cognitive functioning was preserved, as indicated by their scores on the Ravens Standard Progressive Matrices and verbal fluency, which were within the average range for both groups. vmPFC and control patients also had intact verbal and spatial short-term memory, as assessed with the digit span and Corsi tests, and verbal and spatial long-term memory, as assessed with prose recall and recall of the Rey-Osterrieth complex figure. The copy of the Rey-Osterrieth complex figure was also normal. Direct comparison of the vmPFC patients and control patients showed comparable scores on the above neuropsychological tests (*P*’s > 0.10 in all cases).

**Table 1:**
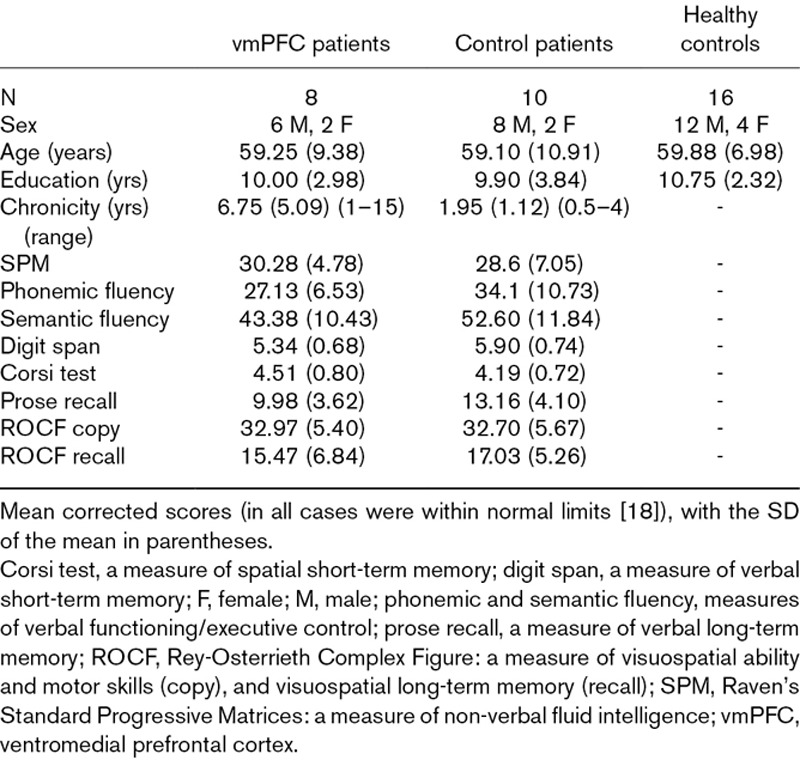
Participants’ demographic and clinical data

Patients were matched to 16 healthy individuals on age (*F*_(2,31)_ = 0.03, *P* = 0.97), education (*F*_(2,31)_ = 0.31, *P* = 0.73) and sex (vmPFC patients: *χ*^*2*^ = 0.00, *P* = 1.00; control patients: *χ*^*2*^ = 0.06, *P* = 0.80). Healthy control participants were not taking psychoactive drugs and were free of current or past psychiatric or neurological illness as determined by history.

All participants gave informed consent in accordance with the local research ethics committees, and in line with the Declaration of Helsinki.

### Stimuli and procedure

**Fig. 2:**
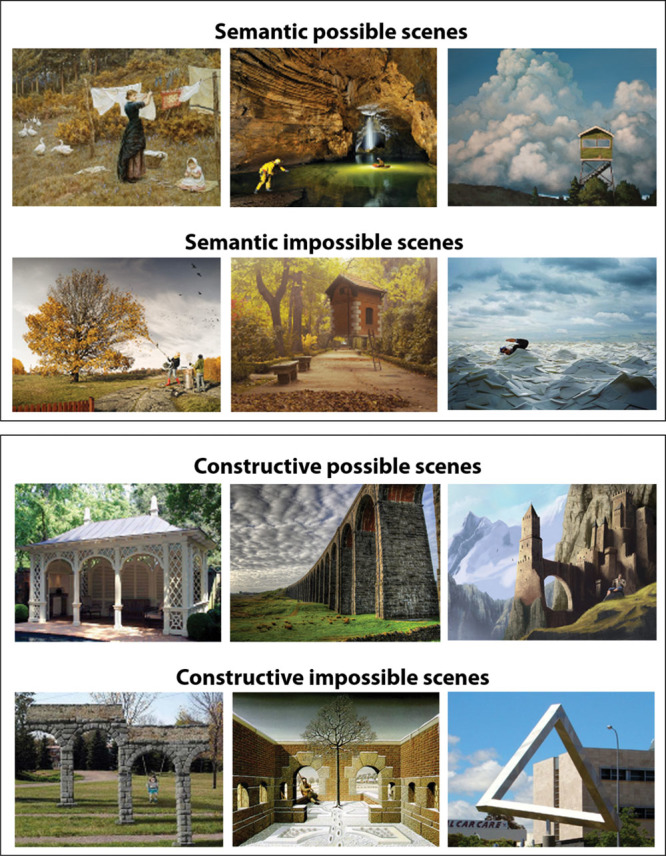
Example stimuli from the possible/impossible scenes task (reproduced from McCormick *et al.* [6]). Semantic scenes are presented in the upper two panels. The possible semantic scene depicts a woman hanging up some laundry, whereas the impossible semantic scene below shows a woman vacuuming the leaves from a tree, which would not happen in the real world. The lower two panels depict examples of constructive scenes. On the left side of the panel, a possible constructive scene includes a typical pavilion, whereas an impossible constructive scene beneath shows arches that would not be possible to build in the real world. Specifically, the top connecting structure suggests a flat architecture, yet the columns of the arches are located at different depths within the scene. Impossible scenes were adapted from the following sources: Semantic: http://www.erikjohanssonphoto.com/; http://www.ucreative.com/inspiration/surreal-photography-of-flying-house-by-rafa-zubiria/; http://www.gettyimages.co.uk/detail/photo/businessman-swimming-in-sea-of-envelopes-high-res-stockphotography/200354836-001; Constructive: http://www.moillusions.com/funny-lookin-arch-illusion/; http://impossible.info/english/art/mey/mey3.html; https://upload.wikimedia.org/wikipedia/commons/3/38/Perth_Impossible_Triangle.jpg.

The stimuli were the same as those used by McCormick *et al.* [6] (Fig. 2). For the semantic violations, the content of an image was wrong in some way (e.g. an elephant with butterfly ears). For constructive violations, an image depicted a spatially implausible scene (e.g. an endless staircase). Participants were not explicitly told whether a picture belonged to the semantic or constructive condition. Each image was presented for 3 seconds at the center of a computer screen, then the question ‘Is this scene possible or impossible?’ appeared underneath it. Participants had up to an additional 20 seconds to look at the scene image and question, and indicated their decision by a key press. Following each possible/impossible decision, participants had up to 15 seconds for each of two ratings: how difficult they found it to decide whether a scene was possible or impossible (1 = not difficult at all, 2 = somewhat difficult, 3 = very difficult); and how confident they were in their decision (1 = not confident at all, 2 = somewhat confident, 3 = very confident).

### Data analyses

Given that in all cases, the dependent variables were normally distributed (Kolmogorov–Smirnov all *d*’s > 0.10, and all *P*’s > 0.05), the data were analysed with parametric tests. Each variable (accuracy, response times, difficulty ratings, confidence ratings) was analysed using a two-way repeated measures analysis of variance with participant group as the between-subjects factor with three levels (vmPFC patients, control patients, healthy controls), and scene category as the within-subject factor with two levels (semantic, constructive).

## Results

**Fig. 3:**
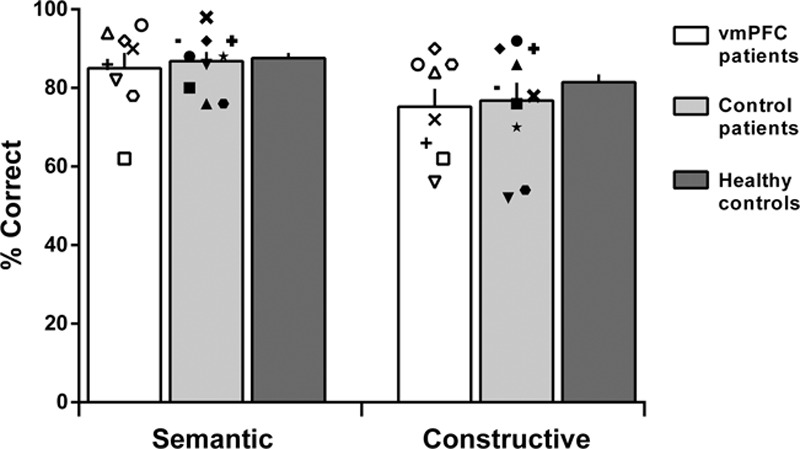
Mean accuracy for vmPFC patients, control patients and the healthy control group for the two scene conditions (semantic, constructive). Symbols represent individual patient performance. Error bars represent the SEM. vmPFC, ventromedial prefrontal cortex.

Table 2 shows the mean scores for each participant group. There were no significant main effects of group or interactions between participant group and scene category for any measure [accuracy (group *F*_(2,31)_ = 0.91, *P* = 0.41; interaction *F*_(2,31)_ = 0.55, *P* = 0.58) (Fig. 3); response times (*F*_(2,31)_ = 0.10, *P* = 0.90; *F*_(2,31)_ = 2.03, *P* = 0.15); difficulty (*F*_(2,31)_ = 0.05, *P* = 0.95; *F*_(2,31)_ = 2.30, *P* = 0.12); confidence (*F*_(2,31)_ = 2.49, *P* = 0.10; *F*_(2,31)_ = 1.43, *P* = 0.25)]. Across groups, there was higher accuracy (*F*_(1,31)_ = 21.34, *P* < 0.001) and shorter response times (*F*_(1,31)_ = 57.23, *P* < 0.001) for the semantic compared to the constructive condition, and decisions about semantic violations were rated as easier (*F*_(1,31)_ = 41.81, *P* < 0.000) and were made more confidently (*F*_(1,31)_ = 50.97, *P* < 0.0001) compared to those for constructive violations.

**Table 2:**
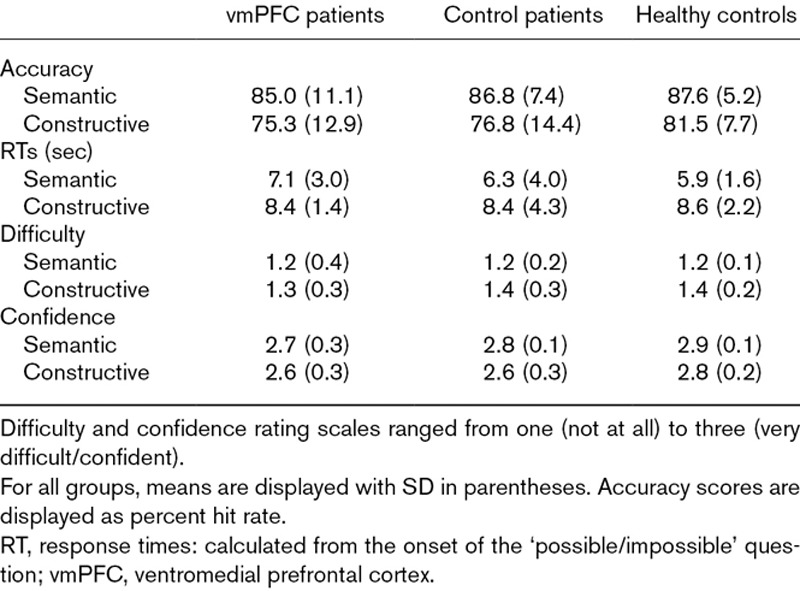
Participants’ mean task performance

## Discussion

Not much is known about scene processing in vmPFC-damaged patients and so our results provide a novel insight into this aspect of their cognition. We found that patients with vmPFC lesions were unimpaired relative to control patients and healthy controls at detecting constructive and semantic violations in naturalistic scene images. Of course, with a null result we cannot conclude definitively that no deficit exists. However, it is notable that a significant impairment on the constructive aspect of this task was reported by McCormick *et al.* [6] in a similar-sized sample of hippocampal-damaged patients.

We also know from McCormick *et al.* [6] that, in particular, the constructive condition seems to require the internal construction of a scene model in order to make a comparison with a physical scene stimulus. This process involves focussing attention inwards, selecting appropriate elements to build an accurate scene model, and then using these components to generate the scene image for comparison. Patients with vmPFC lesions are known to have difficulties with the first two aspects of this pathway [7–10,14], yet they were not impaired on the task. We argue that this is because the scene stimuli, together with the explicit task demands, acted as specific and detailed cues that helped to circumvent their initiation problems. Then, their intact hippocampi were able to generate the necessary scene imagery to perform the final phase of the task. Our findings, therefore, seem to support the view that vmPFC-damaged patients have a preserved ability to construct scene imagery, given their intact hippocampi, and that the vmPFC may implement functions upstream of, and instrumental to, scene construction [12,13].

It is also interesting that the vmPFC-lesioned patients could identify semantic impossibilities in scenes. The vmPFC is thought to play a key role in instantiating superordinate knowledge structures, or schemas [14] that are used to guide memory retrieval or store new information [16,17]. As the discrimination between possible and impossible semantic scenes likely requires the re-activation of memory schemas congruent with the observed scene in order to understand what is typical in a given scenario, an impairment in vmPFC-lesioned patients might have been expected. However, we found that across all participant groups, performance was better for the semantic compared to the constructive scene violations and, therefore, it is possible that the semantic violations in this task are so obvious as to not require reinstatement of detailed knowledge-based schemas [14].

## Conclusion

Our findings help to more precisely characterize the role of the vmPFC in scene processing. We showed that vmPFC-damaged patients are able to perceive scenes accurately, appreciate their spatial-constructive nature, extract meaning and make semantic judgements about them in the presence of very specific cues. This stands in clear contrast to the impairment they display during unconstrained tasks, such as the free recall of autobiographical memories [7], or the imagination of future and fictitious scenarios [7–9]. These situations require the selection of appropriate mental representations or responses, inhibition of those that are competing but irrelevant, using schematic knowledge as a guide.

## Acknowledgements

E.C. is supported by Ricerca Fondamentale Orientata funds from the University of Bologna. E.A.M. is supported by a Wellcome Principal Research Fellowship (210567/Z/18/Z) and the Centre by a Centre Award from Wellcome (203147/Z/16/Z).

## Conflicts of interest

There are no conflicts of interest.
